# Prevalence of the Genes Associated with Biofilm and Toxins Synthesis amongst the *Pseudomonas aeruginosa* Clinical Strains

**DOI:** 10.3390/antibiotics10030241

**Published:** 2021-02-28

**Authors:** Tomasz Bogiel, Dagmara Depka, Mateusz Rzepka, Joanna Kwiecińska-Piróg, Eugenia Gospodarek-Komkowska

**Affiliations:** Microbiology Department, Ludwik Rydygier Collegium Medicum in Bydgoszcz, Nicolaus Copernicus University in Toruń, 85-094 Bydgoszcz, Poland; dagmaradepka@cm.umk.pl (D.D.); mateusz.rzepka@cm.umk.pl (M.R.); j.kwiecinska@cm.umk.pl (J.K.-P.); gospodareke@cm.umk.pl (E.G.-K.)

**Keywords:** biofilm, exotoxins, *Pseudomonas aeruginosa* biofilm, *Pseudomonas aeruginosa* virulence, toxins, virulence, virulence genes

## Abstract

*Pseudomonas aeruginosa* is one of the most commonly isolated bacteria from clinical specimens, with an increasing isolation frequency in nosocomial outbreaks. The hypothesis tested was whether carbapenem-resistant *P. aeruginosa* strains display an altered carriage of the virulence factor genes, depending on the type of carbapenem resistance. The aim of the study was to investigate, by PCR, the frequency of 10 chosen virulence factors genes (*phzM*, *phzS*, *exoT*, *exoY*, *exoU*, *toxA*, *exoS*, *algD*, *pilA* and *pilB*) and the genotype distribution in 107 non-duplicated carbapenem-resistant *P. aeruginosa* isolates. *P. aeruginosa* genes involved in phenazine dyes and exoenzyme T synthesis were noted with the highest frequency (100%). Fimbriae-encoding genes were detected with the lowest incidence: 15.9% and 4.7% for pilin A and B, respectively. The differences observed between the *exoS* gene prevalence amongst the carbapenemase-positive and the carbapenemase-negative strains and the *pilA* gene prevalence amongst the strains of different origins were statistically significant. Virulence genes’ prevalence and the genotype distribution vary amongst *P. aeruginosa* strains resistant to carbapenems, especially in terms of their carbapenemase synthesis ability and the strain origin.

## 1. Introduction

*Pseudomonas aeruginosa* is a ubiquitous Gram-negative non-fermenting rod, a typical bacterial opportunistic pathogen often found in a hospital environment. *P. aeruginosa* strains are responsible for a wide range of human infections, occurring mostly amongst hospitalized patients. Thus, *P. aeruginosa* rods are one of the most often isolated bacteria from clinical specimens. Their increasing resistance to carbapenems and their role in nosocomial outbreaks of different localizations are underlined by their involvement in severe respiratory and urinary tract infections, as well as skin and soft tissue infections or bacteremia [1].

*P. aeruginosa* rods are intrinsically resistant to numerous antimicrobials and also easily acquire antibiotic resistance mechanisms to the majority of antibiotic groups, including carbapenems. These beta-lactam representatives (e.g., imipenem and meropenem) are often the last-resort antibiotics. The enzymatic mechanism of carbapenem resistance usually results from metallo-β-lactamase (MBL) synthesis. The most prevalent of these carbapenemases among *P. aeruginosa* strains worldwide are Verona-Integron-Metallo-beta-lactamases (VIM) and imipenemases (IMP-type enzymes), encoded by *bla*_VIM_ and *bla*_IMP_ genes, respectively. *P. aeruginosa* resistance to carbapenems may also result from a loss of OprD protein porins responsible for carbapenem transport into bacterial cells, overexpression of efflux pumps that actively pump out antimicrobials outside the cell or some other carbapenem-hydrolyzing enzymes. 

It has been previously found, both within our unit as well as other units worldwide, that the incidences of carbapenem-resistant *P. aeruginosa* (CRPA) strain isolation, and their contribution to nosocomial infections, have increased with time [2]. Additionally, it has been shown that CRPA strains may clonally spread worldwide with some dominant sequence types [3].

The pathogenicity of *P. aeruginosa* is based on the synthesis of several cell- and biofilm-associated compounds (e.g., flagella, pili, alginate capsule and lipopolysaccharide). *P. aeruginosa* is also capable of secreting proteins that may play a potential role in its pathogenicity, typically extracellular virulence factors, mostly toxins (e.g., toxin A, exotoxins T, U and Y and exoenzyme S) and enzymes (e.g., elastases and phospholipases) [4]. Exotoxin A, encoded by the *toxA* gene, is responsible for the cytotoxic capacity of *P. aeruginosa*. It is a protein involved in pro-inflammatory cytokine synthesis stimulation. The *algD* gene encodes GDP-mannose 6-dehydrogenase, an enzyme involved in the alginate biosynthesis pathway. This compound is one of the key components of *P. aeruginosa*’s biofilm. The *pilA* and *pilB* genes encode bacterial pilins—the structural proteins synthesized by *P. aeruginosa* which are strongly involved in bacterial adhesion, as well as biofilm formation. The *phzM* and *phzS* genes encode phenazine-1-carboxylate, an enzyme involved in pyocyanin biosynthesis. The role of pyocyanin is to increase the level of reactive oxygen species (ROS) within a eukaryotic cell. An increased concentration of ROS within neutrophils could result in apoptosis of human cells. The *exoS*, *exoT*, *exoU* and *exoY* genes in *P. aeruginosa* encode components of the type III secretion system (TTSS). They are believed to be some of the most important virulence determinants of *P. aeruginosa*.

The above-mentioned virulence factors usually cooperate during *P. aeruginosa* colonization of the host cell and biofilm formation, allowing for further infection and additionally increasing pathogenicity. Most of the virulence factors are chromosomally encoded and their gene sequence has been determined in the *P. aeruginosa* PAO1 strain genome [5]. However, *P. aeruginosa* genome elasticity has been previously confirmed by Ramsay et al. [6].

Although the synthesis of the mentioned virulence factors is controlled by regulatory systems (e.g., bacterial quorum sensing signaling) [4,7], the presence of virulence genes is a key feature of the strains’ pathogenicity. Additionally, it has been shown that the co-existence of antimicrobial resistance and virulence determinants in *P. aeruginosa* isolates is becoming an alarming threat. This underlines the importance of the mentioned issue and demands continuous monitoring of multidrug-resistant (MDR) pathogens in order to determine therapeutic options for the successful treatment of such infections [8].

The aim of this study was to determine the genetic characteristics of 107 non-repeatable CRPA strains by assessing the distribution of two genes encoding MBL (*bla*_VIM_ and *bla*_IMP_) and 10 genes of the virulence factors or the enzymes involved in their biosynthesis (*exoS*, *toxA*, *algD*, *pilA*, *pilB*, *exoT*, *exoY*, *exoU*, *phzM* and *phzS*). The mentioned genes were chosen based on previous studies. An additional purpose of this study was to check whether the virulence potential of CRPA strains differs depending on the type of mechanism of carbapenem resistance.

## 2. Results

### 2.1. The MBL Genes

The prevalence of carbapenem resistance mechanism genes amongst the examined strains was as follows: the *bla*_VIM_ gene was detected in 32 (29.9%) of the examined strains while none of the examined strains were positive for the *bla*_IMP_ gene.

### 2.2. The Virulence Factor Genes

**Figure 1:**
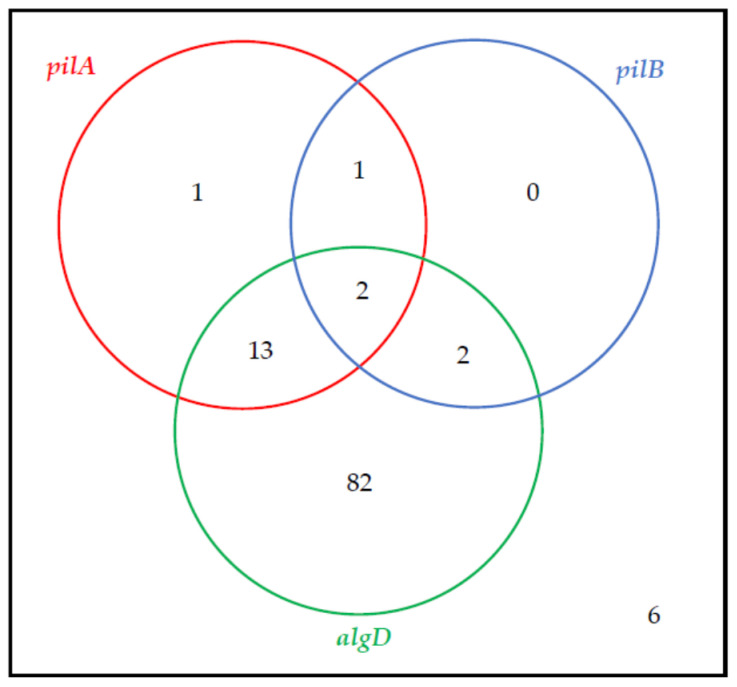
The co-existence of biofilm synthesis-associated genes (Venn diagram) amongst the tested strains (*n* = 107).

**Figure 2:**
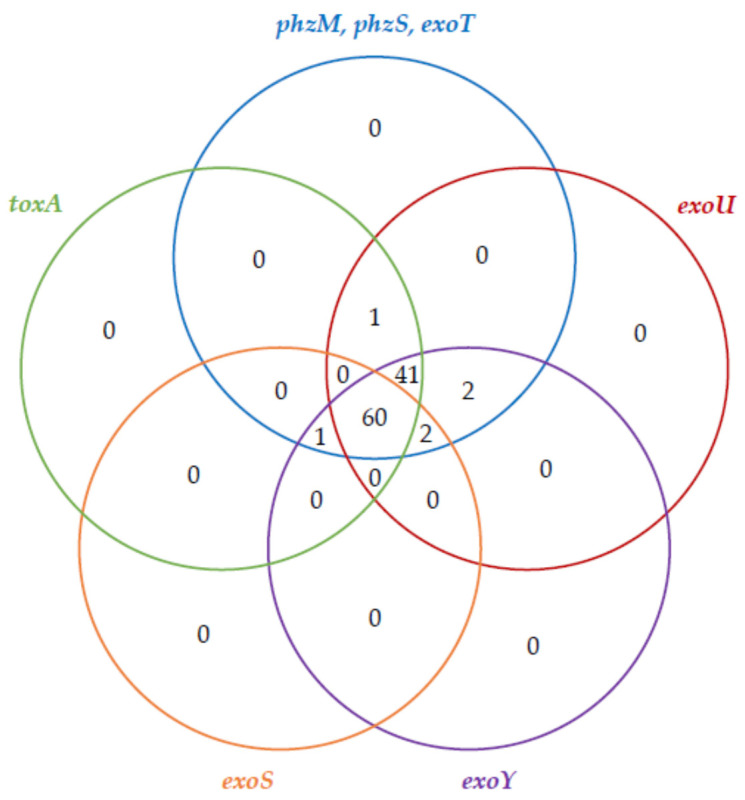
The co-existence of toxin synthesis-associated genes (Venn diagram) amongst the examined *P. aeruginosa* strains (*n* = 107).

Assessment of the virulence background revealed a wide gene distribution variety. The prevalence of virulence genes amongst the examined strains was as follows: the *exoT*, *phzM* and *phzS* genes were noted in all of the tested strains, whereas the *pilA* and *pilB* genes were observed with the lowest frequency—amongst 15.9% and 4.7% of the examined isolates, respectively. The occurrence of the remaining virulence genes was as follows: 99.1% for *exoY* and *exoU*; 96.3%—*toxA*; 92.5%—*algD*; and 58.9% for *exoS*. Venn diagrams representing the co-existence of the biofilm-associated genes and toxin genes amongst the examined *P. aeruginosa* strains are shown in Figure 1 and Figure 2, respectively.

**Figure 3:**
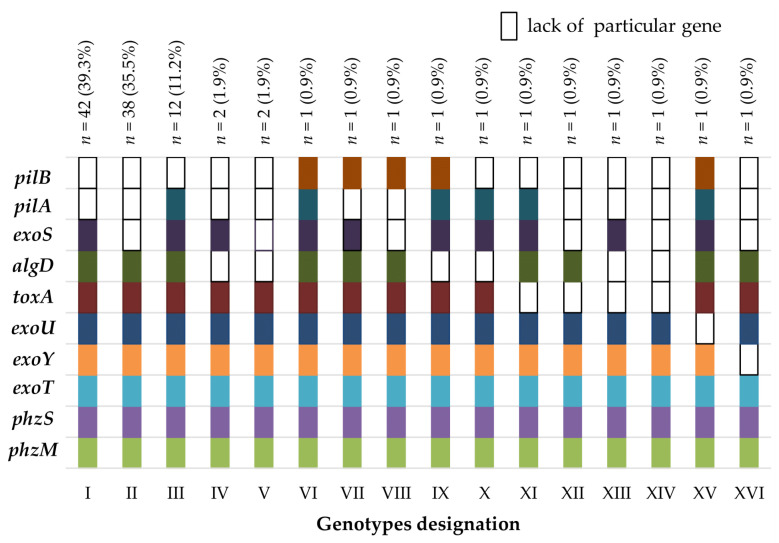
Genotypes’ distribution detected amongst the examined *P. aeruginosa* strains (*n* = 107).

Sixteen observed genotypes (named I–XVI) and their prevalence with the distribution amongst the examined strains are shown in Figure 3. The most prevalent genotype, including all the genes detected (except for the *pilA* and *pilB* genes), was observed among 42 (39.3%) of the isolates. Eleven (10.3%) strains presented distinctive genotypes (numbered VI–XVI). The distribution and co-existence of the genotypes noted amongst *P. aeruginosa* strains derived from surgery, intensive care units (ICU) and rehabilitation clinics are presented in Table S1 and Figure S1. The corresponding genotypes’ distributions and co-existence with respect to the specimens that the strains were isolated from are presented in Table S2 and Figure S2. Of note, genotype II was the most dominant amongst the strains derived from the ICU patients, while for the remaining wards and the whole group of strains included in the study, the most prevalent was genotype I. A statistically significant difference (χ^2^ test) was additionally observed between the *exoS* gene frequency when compared to the rest of the tested genes prevalence (*p* < 0.001).

The differences between the virulence factor genes’ frequency noted between carbapenemase-positive and carbapenemase-negative CRPA strains are indicated in Table 1. The greatest differences in the positive results of these genes in terms of the carbapenemase synthesis ability were observed for the *exoS* gene (77.0% vs 18.2%—statistically significant difference, *p* < 0.001) and the *pilA* gene (20.3% vs 6.1%).

A statistical analysis also revealed a positive correlation for genes’ coexistence, noted for the following pairs of genes: *toxA*/*algD* (0.34471) and *exoS*/*pilA* (0.340511).

Additionally, the *pilA* gene was noted more frequently among the strains derived from gastrointestinal tract infections when compared to the strains isolated from respiratory (0.0250), bloodstream (0.0229) or skin and soft tissue infections (0.0394), and when compared to the *pilB* gene frequency among the strains isolated from the gastrointestinal tract (0.0389).

Moreover, the *pilA* gene was noted less frequently when compared to the *exoS* gene among the strains derived from urinary (0.0094), respiratory tract (<0.0000), bloodstream (0.0184) or skin and soft tissue infections (0.0352). As well as this, the *pilB* gene was noted statistically less frequently than the *exoS* gene, regardless of the clinical specimen type that the strains were isolated from.

Of note, in the genotypes distribution analysis, with respect to the strains’ origin, the only statistically significant relation was observed for the strains with genotype III that were noted more often from the gastrointestinal tract when compared to those of respiratory tract origin.

## 3. Discussion

In recent years, an increased number of carbapenem-resistant strains among *P. aeruginosa* isolates has been observed. Infections due to CRPA are currently one of the most dangerous healthcare-associated infections, since carbapenems are often the last-choice drugs in the treatment of infections with this etiology [9]. In the available literature, numerous researchers have described the genetic features of *P. aeruginosa* with respect to different variables (e.g., strain origin, susceptibility to antimicrobials, clinical specimen and patients’ hospitalization length). However, little information on the virulence gene prevalence and genotype distribution amongst the CRPA strains can be found in the relevant literature [10,11].

In the available literature, the only significant relationship between virulence factors and beta-lactams resistance was observed for the strains with oxacillinases (OXA)- and IMP-like beta-lactamase genes [12]. The association between the CRPA phenotype and virulence genes carriage is still unclear and needs further studies. 

Relatively much is known about the association between *P. aeruginosa* strains type III secretion, their antibiotic resistance and the clinical outcome of the patients infected by them [13]. However, to our knowledge, the only correlation between antibiotic resistance and a specific virulence genotype, in a group of CRPA strains, was observed between the *exoU* gene and the mechanism of resistance to fluoroquinolones [14].

In the present study, the prevalence of the two genes encoding MBL (*bla*_VIM_ and *bla*_IMP_) and 10 genes of the virulence factors or enzymes involved in their biosynthesis (*exoS, toxA, algD, pilA, pilB, exoT, exoY, exoU, phzM* and *phzS*) was evaluated. Interestingly, there was only one strain among the examined isolates group that carried all of the genes for the studied virulence factors. Of note, the mentioned strain was negative for both of the investigated carbapenemase genes. Further studies are needed to decipher the hyper-virulent genetic potential of this particular isolate and its pathogenic properties.

In our study, 32 (29.9%) of the isolates were *bla*_VIM_-positive, while none of the strains were *bla*_IMP_-positive. This finding is quite different from another study carried out in Poland among pediatric patient-derived strains, which showed that only 10.9% (6/55) of *P. aeruginosa* isolates were *bla*_VIM_-positive and 1.8% (1/55) *bla*_IMP_-positive [15]. Our result is also different from the observation noted by Sharifi et al. [16], in which the predominant MBLs (84.5%) were IMP-like enzymes. In the cited study, VIM-like carbapenemases were observed amongst 4.8% of the investigated strains only. In turn, Pobiega et al. obtained similar results to ours for CRPA strains isolated from patients with urinary tract infections (UTIs) in southern Poland [17]. Interestingly, in different countries, *P. aeruginosa* carrying the *bla*_IMP_ gene is also more prevalent than that with *bla*_VIM_ [18,19,20]. Since the pathogenic strains are derived mainly from hospitalized patients, these results could confirm an endemic intra-hospital spread of this species.

The resistance to carbapenems among *P. aeruginosa* may also be conditioned by other mechanisms (e.g., loss of OprD protein, overexpression of efflux pumps and other carbapenem-hydrolyzing enzymes) [9]. Therefore, the mechanism of the antibiotic resistance type, resulting from *bla*_VIM_ gene presence, was found only amongst 29.9% of the strains. The genetic background (e.g., lack of OprD porins or efflux pumps) of the carbapenem resistance among the remaining strains needs further investigation.

In this study, the frequency of the chosen 10 virulence factor genes in CRPA was examined and the strains selected for this study show a relatively high percentage of virulence factor genes. One of the most common *P. aeruginosa* virulence factor genes detected in our study, amongst 103 (96.3%) analyzed strains, was the *toxA* gene. To our knowledge, the only research on *toxA* gene carriage among CRPA was conducted by Goncalves et al. [11]. The mentioned authors obtained similar results—in their study, 87.5% of the CRPA strains carried the *toxA* gene. Other researchers who analyzed *P. aeruginosa* isolates derived from respiratory tract infections, but not CRPA exclusively, also showed a high percentage of the *toxA* gene (80.3%) [21]. Our results confirm the observation revealed in previous studies: the *toxA* gene is more common among MDR strains and, thus, may lead, additionally, to their increased virulence [22]. Noteworthily, a positive correlation was observed for the *toxA* and *algD* genes’ existence, suggesting that there might be a relationship between the carriage of toxins and biofilm genes in *P. aeruginosa*, but this observation requires further studies.

The majority of pathogenic *P. aeruginosa* are capable of biofilm formation. As a result, they tend to be more difficult to remove from the hospital environment, as well as from medical and diagnostic equipment. This virulence determinant is common in *P. aeruginosa* strains [8]. To our knowledge, the only phenotypic study on biofilm-involved virulence factors of CRPA was conducted by El-Mahdy and El-Kannishy [23]. The mentioned property was found amongst 77.5% of the examined strains. In our study on the *algD* gene’s presence, 99 (92.5%) CRPA strains were positive. These results are consistent with the results of the research carried out by Ellappan et al. [8], showing that 92.9% of CRPA strains carry the *algD* gene. The percentage of the *algD* gene reached 87.5% in a group of *P. aeruginosa* isolates (not CRPA exclusively) examined by Kamali et al. [24], with 88.1% and 84.6% for biofilm producer and non-biofilm strains, respectively.

The second determinants of bacterial biofilm formation are pilins. However, relatively low percentages of the *pilA* (15.9%) and the *pilB* (4.7%) genes were observed among the studied *P. aeruginosa* strains. These results are comparable with the second research study from Poland among *P. aeruginosa* strains derived from patients with a UTI (not CRPA exclusively). In their study, 19.2% of the isolates carried the *pilA* gene, while none of the strains were positive for the *pilB* gene [17]. Another study showed that the percentage of the *pilB* gene reached 8.3% in a group of MDR *P. aeruginosa* isolates, examined by Sharifi et al. [16]. Only three of the strains studied in the present work carried both of the mentioned genes. These results indicate that for the evaluated strains group, the potential for alginate synthesis, observed at least on a molecular level, might be one of the major determinants involved in biofilm formation. Interestingly, a positive correlation was observed for the *pilA* and *exoS* genes’ co-existence. 

*pilA* gene carriage was noted most frequently among the strains derived from the gastrointestinal tract when compared to strains of other origin. In our opinion, this finding is very interesting, suggesting that this particular gene’s presence is crucial for human gastrointestinal tract colonization or infection. Thus, there may be a potential relationship between the selected virulence genes’ carriage and *P. aeruginosa*’s ability to form biofilm or colonize a particular place, but this finding needs further studies.

All of the investigated CRPA isolates were positive for the *phzM* and *phzS* genes (*n* = 107). This may suggest that pyocyanin is one of the most prevalent virulence determinants of CRPA. Noteworthily, Fuse et al. [25] showed that in a group of MDR *P. aeruginosa* strains, the synthesis of pyocyanin decreases. Since our study results show that all of the CRPA strains carry the *phzM* and *phzS* genes, it is possible that either the decreased production of pyocyanin is associated with resistance to other groups of antibiotics or that its synthesis is more likely controlled at the expression level, not carriage itself [25]. It has also been previously shown that there is a significant difference in the *phzS* gene’s presence between environmental and clinical *P. aeruginosa* strains [26]. Noteworthily, in the relevant literature, the first mentioned group is considered as more sensitive to antimicrobials, while the second one—more resistant or even multidrug-resistant [26].

In our study, a relatively high prevalence of the TTSS genes (*exoT*—100%; *exoU* and *exoY*—99.1%; *exoS*—58.9%) was noted. The data from a recent meta-analysis on *P. aeruginosa* isolates with toxin potency indicated relatively high prevalence of exotoxins in *P. aeruginosa* clinical isolates. In turn, the ability to synthesize toxins is of fundamental importance from a clinical aspect. It was found that the *exoT* gene had the highest prevalence rate among *P. aeruginosa* exotoxins (0.83 (CI95%: 0.64–0.96)), reaching up to 100% amongst CRPA isolates exclusively [27]. It is consistent with the results of the present study.

The results of the *exoS* gene presence in our study are also concordant with the observations made by Khodayary et al. [28] and relatively close to those of Sharifi et al. [16], in which the percentage of the *exoS* gene in a group of the examined MDR *P. aeruginosa* isolates (but not for CRPA exclusively) reached 59.0% and 44.0%, respectively. The statistically significant difference observed in the *exoS* gene incidence in terms of carbapenemase synthesis, as the only TTSS gene, is an interesting and unclear finding that also needs further research.

Khodayary’s study also showed a high frequency of TTSS genes among MDR *P. aeruginosa* strains—all of the strains possessed the *exoT* and *exoY* genes, while the *exoU* gene was noted among 41.0% of the isolates [28]. As previously indicated, the occurrence of a particular TTSS gene is associated with increased resistance to antibiotics [28], at least amongst the strains derived from burn patients. It was even proposed that the mentioned association of MDR and the presence of specific virulence genes might be a predictive marker for a worse clinical condition of the infected patients [28].

Our study results also showed that among *bla*_VIM_-positive isolates, 81.8% (*n* = 27) presented the *exoU*+/*exoS*− genotype. It has also been shown that the *exoU*+/*exoS*− genotype predominates in a high-risk *bl*a_VIM_-positive ST235 clone [29], which is the most disseminated clone worldwide [30]. Further studies would be necessary to confirm the mentioned strains’ subgroup classification to the most prevalent sequence type.

The results of the present study show, in turn, that among the *bla*_VIM_-negative isolates (*n* = 74), 57 carried the *exoS* gene. Only one strain carrying the *exoU*−/*exoS*+ genotype was negative for the *bla*_VIM_ gene.

In the relevant literature, the association between antimicrobial resistance (or MDR phenotype) and genes carriage was investigated the most in terms of the *exoU* gene. As it has been previously indicated, the occurrence of this particular gene is associated with increased resistance to some antibiotics [28,31], and the *exoU* gene is more often found amongst MDR *P. aeruginosa* strains [32]. On the contrary, the *exoU* gene’s presence reached only 26.0% in a group of MDR *P. aeruginosa* isolates examined by Sharifi et al. [16]. These discrepancies may also result from the different definitions of multi-drug resistance applied by different researchers.

To summarize, higher antimicrobial resistance is generally observed in *P. aeruginosa* isolates possessing the *exoU* gene. The researchers propose that understanding the prevalence of the *exoU*-positive and the mRNA overexpression of resistance genes may help to select empirical therapy for the treatment of nosocomial infections caused by *P. aeruginosa* [31]. Noteworthily, 99.1% of the carbapenem-resistant isolates in this study also carried the *exoU* gene, and this finding is concordant with the results of previous research. In the meta-analysis on *P. aeruginosa* toxin genes, the lowest prevalence for the *exoU* gene reached 0.32 (CI95%: 0.24–0.41) [27].

In summary, a great genetic diversity is observed amongst CRPA strains. This variability is based on the existence of a particular plasmid or chromosomal genes set. The cause of this diversity might be the genetic material organization or “management”—the property of attaining and conserving the genes crucial for survival in the hospital environment (e.g., genes of carbapenem-resistance) and losing the currently less important virulence factor genes (e.g., pilin-encoded genes). However, the mentioned properties can also be achieved through the particular expression of relevant genes. As it has been previously shown, massive changes in the transcriptome of the strains are observed [33], including metabolic changes and the alteration of virulence—for example, during bloodstream infections [34]. These results suggest that, as part of its survival strategy and in response to changes in the patients’ blood, *P. aeruginosa* enhances the expression of certain virulence genes and reduces the expression of others [33,34].

Taken together, this study is believed to be one of the first and largest evaluations of virulence factor genes’ carriage in a group of CRPA strains exclusively. It reports on the diverse virulence potential of *P. aeruginosa* strains, at least on a genetic level, also with respect to carbapenemases synthesis ability. Further research is required to understand the mechanism of *P. aeruginosa* virulence genes’ carriage and the involvement of other contributing factors to this phenomenon, e.g., the mentioned genes’ expression evaluation, as previously shown [35].

The results of the simultaneous evaluation of exotoxins and antimicrobial resistance can be used to develop treatment policies against *P. aeruginosa* infections in hospitals and amongst hospitalized patients [27]. In effect, it would provide the necessary tools for the rapid diagnosis of hyper-virulent strains, as well as allowing us to monitor their spread. It could, finally, help to establish better strategies for the treatment [36,37] or control of infections caused by *P. aeruginosa.* For example, searching for the quorum sensing inactivation compounds [38,39,40] and selected virulence gene expression (down)regulation [40,41,42] may be achieved either by using natural agents [39,40,43] or attempting to increase the strains’ sensitivity to existing antimicrobials [44].

## 4. Materials and Methods

### 4.1. Origin of Strains and Selection Criteria

In total, 107 clinical isolates of imipenem- and meropenem-resistant *P. aeruginosa* strains were included in the study. The strains were initially identified based on typical growth on a selective medium (Cetrimide agar, *bio*Mérieux, France) and biochemical reaction results. The final identification was made using a MALDI-TOF MS device (Bruker, Mannheim, Germany). All the strains were also initially screened for the presence of MBLs, OXA-like and *Klebsiella pneumoniae* carbapenemases (KPC) enzymes (data not shown). All of them were isolated in the Clinical Microbiology Laboratory of Dr Antoni Jurasz University Hospital No. 1 in Bydgoszcz, Poland. Most of the strains were derived from patients in the Anesthesiology and Intensive Care Unit (45–42.1%), different surgery clinics (26–24.3%) as well as the Rehabilitation Ward (16–15.0%). Each strain was isolated from a different patient. All the strains were also initially tested genotypically using the polymerase chain reaction—random amplified polymorphic DNA (PCR-RAPD) technique to exclude repeated isolates [45]. According to Mahenthiralingam et al.’s methodology, PCR-RAPD primers 208 and 272 were applied to confirm the absence of duplicate strains in the further study and analysis. The strains with an identical PCR-RAPD genotypic pattern were excluded from the further study (data not shown). The strains included in the study were isolated mostly from respiratory tract- (31, 29.0%), urinary tract- (25, 23.4%), wounds- (21, 19.6%), bloodstream- (12, 11.2%) and gastrointestinal tract-derived (10, 9.3%) specimens.

### 4.2. The Susceptibility to Carbapenems and Carbapenemase Activity Evaluation

During the antimicrobial susceptibility testing step, the following were used: the diffusion method on Mueller–Hinton agar (Becton Dickinson, Germany), imipenem (10 µg) and meropenem (10 µg) discs (Becton Dickinson, Germany) as well as Etest^®^ strips (*bio*Mérieux, France). The results of the antimicrobial susceptibility tests were interpreted according to the European Committee on Antimicrobial Susceptibility Testing recommendations (EUCAST, Breakpoint tables for bacteria, Clinical breakpoints—bacteria v 9.0). *P. aeruginosa* obtained from the American Type Culture Collection (ATCC 27853) as well as *Escherichia coli* ATCC 25922 strains served as a susceptibility testing quality control.

All of the examined strains were also checked phenotypically for MBL synthesis, using the method described by Lee et al. [46] and by Yong et al. [47]. Among the examined strains, only the *bla*_VIM_ gene-positive isolates were positive for the detection of an enzymatic resistance mechanism to carbapenems, regardless of the methodology applied (data not shown). For all of the strains with positive carbapenemases tests, VIM-like enzymes were detected. Of note, this particular type of enzymatic resistance to carbapenems is the only mechanism recognized among the *P. aeruginosa* collection from our department. They were subsequently called carbapenemase-positive, while the rest were noted as carbapenemase-negative.

### 4.3. Bacterial DNA Isolation

A Genomic Mini kit (A&A Biotechnology, Gdynia, Poland) was applied for the DNA isolation, according to the manufacturer’s protocol. The DNA samples were stored at 4 °C before their further use for the purpose of PCR.

### 4.4. MBL Genes Detection

PCR was performed to detect the genes for the most common carbapenemases in Poland (VIM- and IMP-like enzymes) in the duplex version, as previously described [48]. The PCR reaction products were separated by electrophoresis on 1% agarose gel in a 1 × Tris-Boric Acid-EDTA (TBE) running buffer at 9 V/cm for one hour in a MINI SUB^TM^ DNA CELL (BioRad, Feldkirchen, Germany) device. Their pictures were recorded and documented in Gel Doc 2000 system using the Quantity One (BioRad, Feldkirchen, Germany) program. *P. aeruginosa* strains carrying the *bla*_IMP_ or *bla*_VIM_ genes served as a positive control, while *P. aeruginosa* ATCC 27853 as a negative control, of the PCR (Figure S3).

### 4.5. Virulence Factor Genes Detection

The prevalence of 10 virulence factor genes was determined by PCR, in a separate reaction for each gene. The genes were amplified with primers selected on the basis of the published PAO1 strain genome sequence and the *P. aeruginosa* ATCC 27853 isolate. The amplification procedure was carried out as previously described [49,50]. The primers’ sequences and the PCR annealing temperatures for each gene amplification are presented in Table 2. The DNA isolated from the *P. aeruginosa* PAO1 strain (kindly provided by the National Medicines Institute in Warsaw, Poland) and the *P. aeruginosa* ATCC 27853 isolate served as a PCR positive control. In the amplification procedure, a thermal cycler GeneAmp^®^ PCR System 2700 (Applied Biosystems, Foster City, CA, USA) was applied.The presence of amplicons for the particular genes was evaluated visually with the application of gel electrophoresis, based on the product size and control strain DNA amplification (Figures S4–11). The duplicates of PCR were performed for the selected strain–gene pairs to confirm the repeatability of the results, giving the consent results in each case (data not shown). 

### 4.6. Statistical Methods

Statistical analysis was performed in the StatSoft Inc. (2017) STATISTICA 13.1 (data analysis software system) program using the standard chi-square test (χ^2^) with α ≤ 0.05 to determine the significance of the differences between the investigated genes’ prevalence amongst the examined strains and the genes’ distribution in the two distinct examined strain subgroups (carbapenemase-positive and carbapenemase-negative). The chi-square test (with Yates’ correction for small groups tested) was applied to investigate the differences in the distribution of the virulence genes and genotypes with respect to the origin of the strains. The Spearman’s rank-order correlation test was applied to investigate the association of the particular genes’ co-existence (α ≤ 0.05).

## 5. Conclusions

High genetic diversity was noted amongst the CRPA strains—all or most of the strains possess genes involved in phenazine synthesis and the genes for the exoenzymes released in the TTSS. Genes encoding *P. aeruginosa* pilins were found at the lowest frequency, suggesting that, at least for this particular strain group, alginate synthesis is a more likely factor of biofilm-associated virulence. The prevalence of the *exoS* gene varied the most with respect to the ability of carbapenemase synthesis. It is likely that the decreased virulence of CRPA strains, at least in some cases, may result from a particular gene loss, especially for carbapenemase producers.

## Figures and Tables

**Table 1:** The prevalence of the virulence factor genes with respect to the presence of carbapenem resistance mechanisms (*n* = 107); (*)—a statistically significant difference (χ^2^ test) noted between the *exoS* gene frequency in the two strain subgroups is indicated (*p* < 0.001).

Subgroup	Gene	*phzM*	*phzS*	*exoT*	*exoY*	*exoU*	*toxA*	*algD*	exoS *	*pilA*	*pilB*
**Carbapenemase (−)**	***n* = 74**	74	74	74	73	73	72	69	57	15	4
	**%**	100.0	100.0	100.0	98.6	98.6	97.3	93.2	77.0	20.3	5.4
**Carbapenemase (+)**	***n* = 33**	33	33	33	33	33	31	30	6	2	1
	**%**	100.0	100.0	100.0	100.0	100.0	93.9	90.9	18.2	6.1	3.0

**Table 2:** The PCR primers’ specification and the PCR parameters applied in the present study.

Gene Detected	PCR Primer Name	Manufacturer	Primer Sequence 5′→3′	Tm (°C)	Annealing Temperature (°C)	Product Size (bp)
***phzS***	phzS F	Sigma	TCGCCATGACCGATACGCTC	55.9	63	1752
	phzS R		ACAACCTGAGCCAGCCTTCC	55.9		
***exoT***	exoT F		AATCGCCGTCCAACTGCATGCG	58.6	64	152
	exoT R		TGTTCGCCGAGGTACTGCTC	55.9		
***exoY***	exoY F		CGGATTCTATGGCAGGGAGG	55.9	64	289
	exoY R		GCCCTTGATGCACTCGACCA	55.9		
***exoU***	exoU F		CCGTTGTGGTGCCGTTGAAG	55.9	64	134
	exoU R		CCAGATGTTCACCGACTCGC	55.9		
***toxA***	toxA F	Integrated DNA Technologies	GGTAACCAGCTCAGCCACAT	57.4	52	352
	toxA R		TGATGTCCAGGTCATGCTTC	54.8		
***algD***	algD F		ATGCGAATCAGCATCTTTGGT	55.2	50	1310
	algD R		CTACCAGCAGATGCCCTCGGC	62.5		
***exoS***	exoS F		CTTGAAGGGACTCGACAAGG	55.2	53	504
	exoS R		TTCAGGTCCGCGTAGTGAAT	56.2		
***phzM***	phzM F	Genomed	ATGGAGAGCGGGATCGACAG	55.9	54	875
	phzM R		ATGCGGGTTTCCATCGGCAG	55.9		
***pilA***	pilA F		ACAGCATCCAACTGAGCG	50.3	59	1675
	pilA R		TTGACTTCCTCCAGGCTG	50.3		
***pilB***	pilB F		TCGAACTGATGATCGTGG	48.0	56	408
	pilB R		CTTTCGGAGTGAACATCG	48.0		

## Data Availability

The data presented in this study are available on request from the corresponding author.

## Supplementary Materials

The following are available online at https://www.mdpi.com/2079-6382/10/3/241/s1, Figure S1: Coexistence of particular genotypes (Venn diagram) noted amongst *P. aeruginosa* strains derived from surgery, ICU and rehabilitation clinics; Figure S2: Co-existence of particular genotypes (Venn diagram) noted amongst *P. aeruginosa* strains derived from clinical specimens collected for the diagnostics of respiratory tract infection (RTI), urinary tract infection (UTI), gastrointestinal tract infections (GTI), bloodstream infection (BSI) and skin and soft tissue infection (SSTI); Figure S3: Picture of an electrophoretic gel showing the amplicons of duplex PCR for MBL genes; Figure S4: Picture of an electrophoretic gel showing the amplicons of PCR for the *algD* gene; Figure S5: Picture of an electrophoretic gel showing the amplicons of PCR for the *exoS* gene; Figure S6: Picture negative of an electrophoretic gel showing the amplicons of PCR for the *exoT*, *exoU* and *exoY* genes; Figure S7: Picture of an electrophoretic gel showing the amplicons of PCR for the *phzM* gene; Figure S8: Picture of an electrophoretic gel showing the amplicons of PCR for the *phzS* gene; Figure S9: Picture of an electrophoretic gel showing the amplicons of PCR for the *pilA* gene; Figure S10: Picture of an electrophoretic gel showing the amplicons of PCR for the *pilB* gene; Figure S11: Picture of an electrophoretic gel showing the amplicons of PCR for the *toxA* gene. Table S1: Distribution of the genotypes noted amongst *P. aeruginosa* strains derived from surgery, ICU and rehabilitation clinics; Table S2: Distribution of the genotypes noted amongst *P. aeruginosa* strains derived from clinical specimen collected for the diagnostics of respiratory tract infection (RTI), urinary tract infection (UTI), gastrointestinal tract infection (GTI), bloodstream infection (BSI) and skin and soft tissue infections (SSTI).
