# Supplementary material for: Prevalence of the Genes Associated with Biofilm and Toxins Synthesis amongst the Pseudomonas aeruginosa Clinical Strains
Source: Antibiotics (Basel). 2021 Feb 28;10(3):241. doi: 10.3390/antibiotics10030241 (PMC7997207; doi:10.3390/antibiotics10030241)
Supplement: Supplementary file 1 [file antibiotics-10-00241-s001.pdf]

Table S1. Distribution of the genotypes noted amongst *P. aeruginosa* strains derived from surgery, ICU and rehabilitation clinics

Strain origin	Genotype No.	n	%
Surgery Clinics (n=26)	I	11	42.3
	II	6	23.1
	III	5	19.2
	V	1	3.8
	VI	1	3.8
	X	1	3.8
	XII	1	3.8
ICU (n=45)	II	21	46.7
	I	14	31.1
	III	5	11.1
	IV	1	2.2
	VIII	1	2.2
	XI	1	2.2
	XIII	1	2.2
	XIV	1	2.2
Rehabilitation Clinic (n=16)	I	8	50.0
	II	4	25.0
	III	1	6.3
	IV	1	6.3
	IX	1	6.3
	XVI	1	6.3

Figure S1. Coexistence of particular genotypes (Venn diagram) noted amongst *P. aeruginosa* strains derived from surgery, ICU and rehabilitation clinics (I-XVI – genotypes designation; (n) – number of strains with a particular genotype)

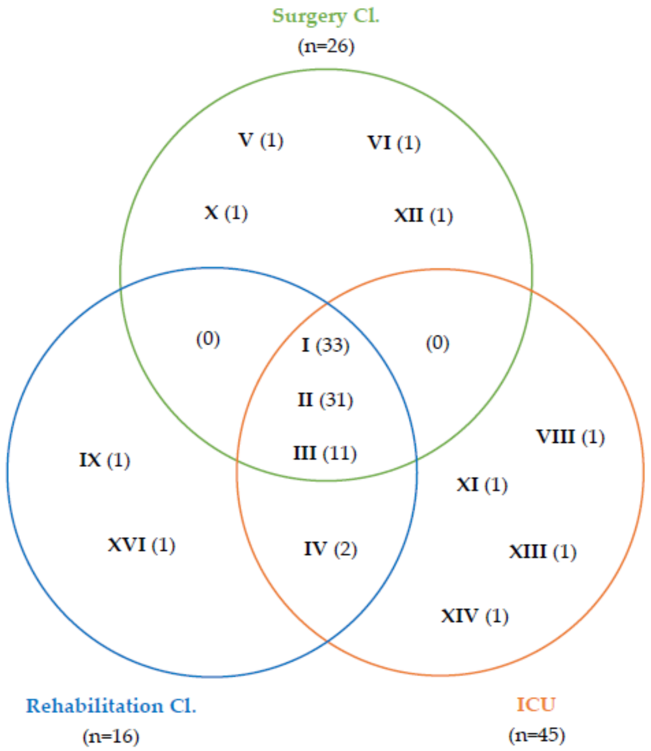

Table S2. Distribution of the genotypes noted amongst *P. aeruginosa* strains derived from clinical specimen collected for the diagnostics of respiratory tract infection (RTI), urinary tract infection (UTI), gastrointestinal tract infections (GTI), blood stream infections (BSI), skin and soft tissue infections (SSTI)

Specimen origin	Genotype No.	n	%
RTI (n=35)	I	18	51.4
	II	10	28.6
	III	2	5.7
	VI	1	2.9
	VII	1	2.9
	XI	1	2.9
	XIV	1	2.9
	XVI	1	2.9
UTI (n=25)	I	9	36.0
	II	8	32.0
	III	3	12.0
	IV	1	4.0
	VIII	1	4.0
	IX	1	4.0
	XII	1	4.0
	XV	1	4.0
GTI (n=10)	III	4	40.0
	II	3	30.0
	I	2	20.0
	X	1	10.0
BSI (n=12)	II	5	41.7
	I	4	33.3
	IV	1	8.3
	V	1	8.3
	XIII	1	8.3
SSTI (n=21)	II	11	52.4
	I	7	33.3
	III	2	9.5
	V	1	4.8

Figure S2. Co-existence of particular genotypes (Venn diagram) noted amongst *P. aeruginosa* strains derived from clinical specimen collected for the diagnostics of respiratory tract infection (RTI), urinary tract infection (UTI), gastrointestinal tract infections (GTI), blood stream infections (BSI), skin and soft tissue infections (SSTI); (I-XVI – genotypes designation; (n) – number of strains with a particular genotype)

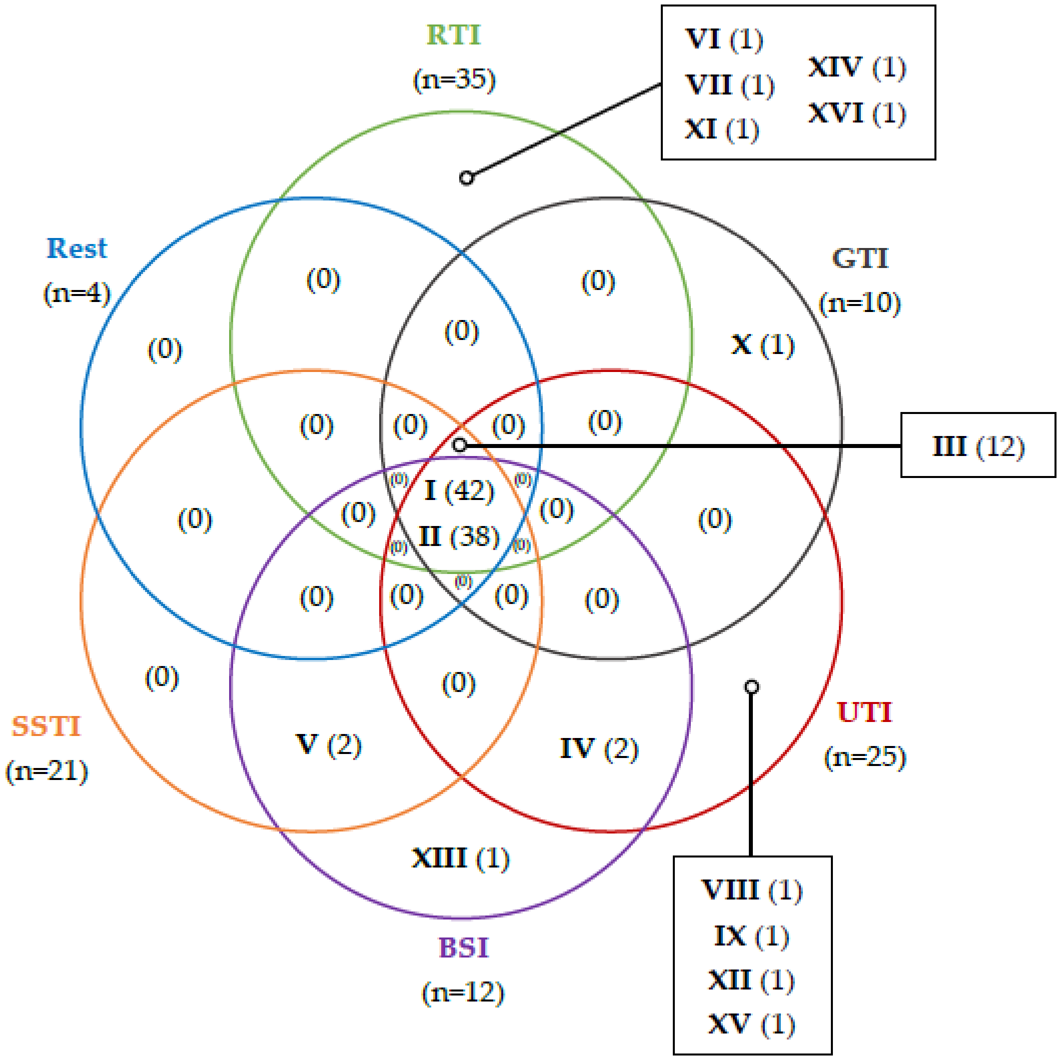

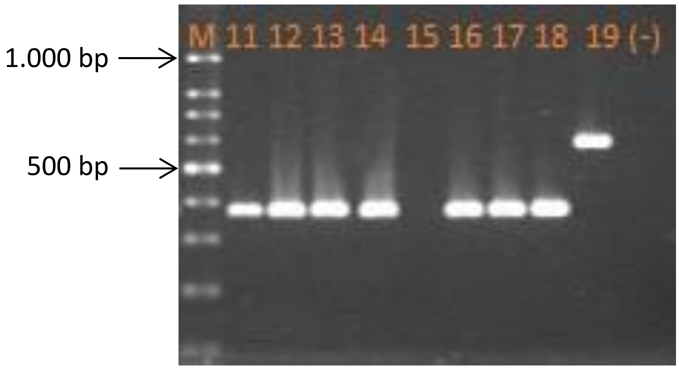

Figure S3. Picture of an electrophoretic gel showing the amplicons of duplex PCR for MBLs genes (M - DNA size marker of 100-1.000 bp; 1-17 - numbers of the examined strains; 18 - bla_{VIM} gene positive control, 19 - bla_{IMP} gene positive control; (-) - negative PCR control)

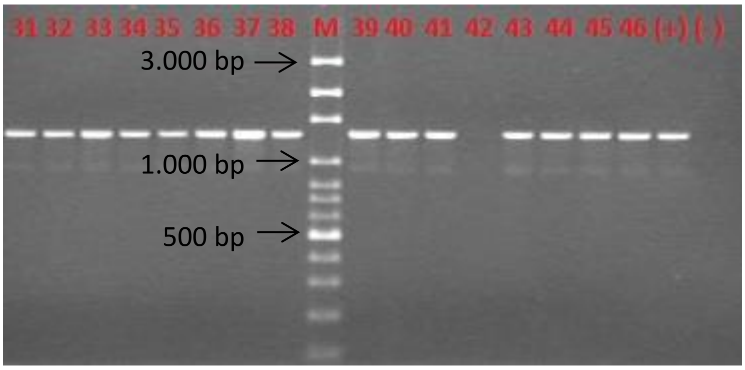

Figure S4. Picture of an electrophoretic gel showing the amplicons of PCR for the *algD* gene (M - DNA size marker of 100-3.000 bp; 31-46 - numbers of the examined strains; (+) - *algD* gene positive control; (-) - negative PCR control)

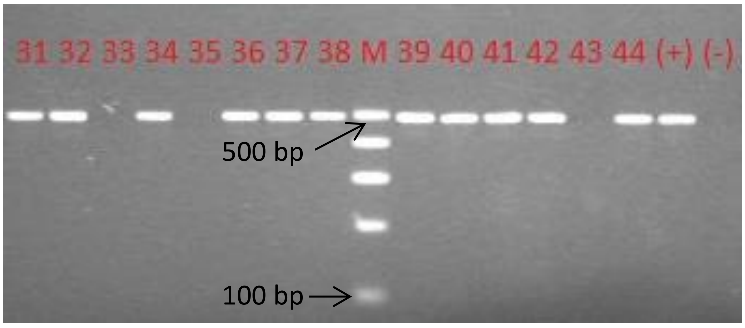

Figure S5. Picture of an electrophoretic gel showing the amplicons of PCR for the *exoS* gene (M - DNA size marker of 100-500 bp; 31-44 - numbers of the examined strains; (+) - *exoS* gene positive control; (-) - negative PCR control)

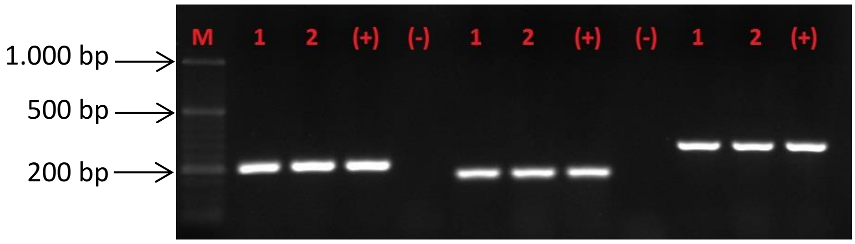

Figure S6. Picture negative of an electrophoretic gel showing the amplicons of PCR for the *exoT*, *exoU* and *exoY* genes (M - DNA size marker of 100-1.000 bp; 1-2 - numbers of the examined strains; (+) - *exoT*, *exoU* and *exoY* genes positive control, respectively; (-) - negative PCR control)

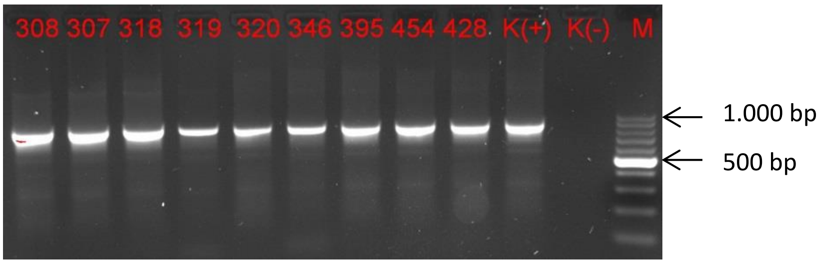

Figure S7. Picture of an electrophoretic gel showing the amplicons of PCR for the *phzM* gene (M - DNA size marker of 100-1.000 bp; 308-428 - numbers of the examined strains; K(+) - *phzM* gene positive control; K(-) - negative PCR control)

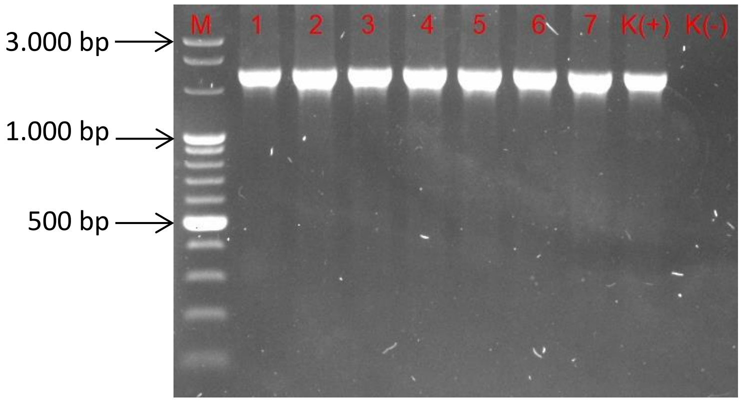

Figure S8. Picture of an electrophoretic gel showing the amplicons of PCR for the *phzS* gene (M - DNA size marker of 100-3.000 bp; 1-7 - numbers of the examined strains; K(+) - *phzS* gene positive control; K(-) - negative PCR control)

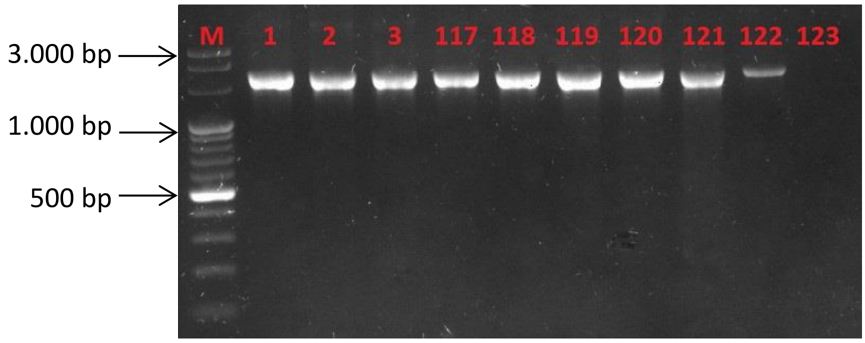

Figure S9. Picture of an electrophoretic gel showing the amplicons of PCR for the *pilA* gene (M - DNA size marker of 100-3.000 bp; 1-3, 117-121 - numbers of the examined strains, 122 – *pilA* gene positive control; 123 - *pilA* gene negative control)

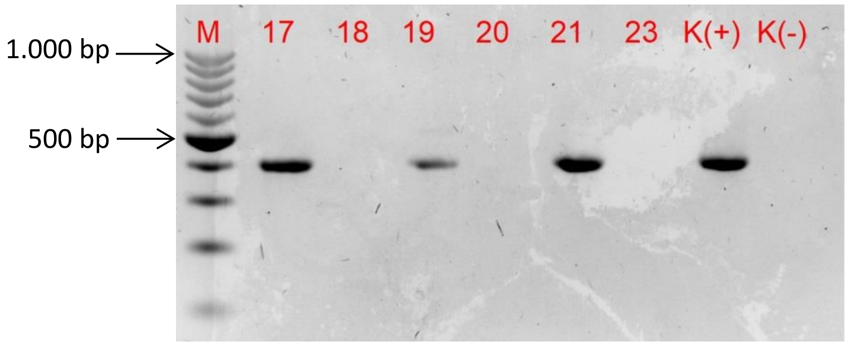

Figure S10. Picture of an electrophoretic gel showing the amplicons of PCR for the *pilB* gene (M - DNA size marker of 100-1.000 bp; 17-23 - numbers of the examined strains, K(+) - *pilB* gene positive control; K(-) - negative PCR control)

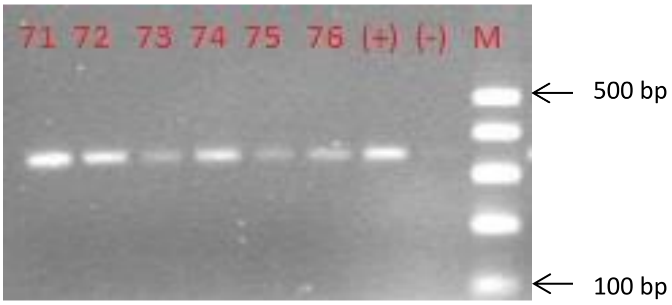

Figure S11. Picture of an electrophoretic gel showing the amplicons of PCR for the *toxA* gene (M - DNA size marker of 100-500 bp; 71-76 - numbers of the examined strains, (+) - *toxA* gene positive control; (-) - negative PCR control)
